# Simultaneous speciation of chromate, molybdate and arsenate in lysimetric water from geotechnical composites installed in field lysimeters

**DOI:** 10.1038/s41598-022-19600-y

**Published:** 2022-09-07

**Authors:** Marija Djurić, Lucija Levstek, Primož Oprčkal, Ana Mladenovič, Alenka Mauko Pranjić, Janez Ščančar, Radmila Milačič

**Affiliations:** 1 Slovenian National Building and Civil Engineering Institute, Dimičeva 12, 1000 Ljubljana, Slovenia; 2 Department of Environmental Sciences, Jožef Stefan Institute, Jamova 39, 1000 Ljubljana, Slovenia; 3 Jožef Stefan International Postgraduate School, Jamova 39, 1000 Ljubljana, Slovenia

**Keywords:** Environmental sciences, Chemistry, Engineering

## Abstract

Anion-exchange high performance liquid chromatography inductively coupled plasma mass spectrometry (HPLC-ICP-MS) was used for simultaneous speciation of chromate, molybdate and arsenate. The repeatability of measurement tested for multielemental standard solution of chromate, molybdate and arsenate (50 ng mL^−1^ of Cr, Mo and As, pH 12) was ± 0.9%, ± 4.9% and ± 4.1%, respectively. Limits of quantification (LOQs) were low (0.53 ng mL^−1^ for chromate and arsenate and 1.03 ng mL^−1^ for molybdate, expressed as elemental concentrations). A wide linear concentration range (from LOQs to 500 ng mL^−1^) was obtained. The performances of this method enabled simultaneous speciation analysis in samples of water from lysimeters, in which three geotechnical composites, made of recycled waste, were installed in parallel in compacted and uncompacted, 20 times less dense form. The release of toxic chemical species of elements into lysimetric waters from each composite was studied. The results revealed that the degree of compaction and the composition of composites both have a significant influence on leaching of chromate, molybdate and arsenate. The study proved that multielemental speciation analysis is fast and cost-effective method for investigations of environmental impacts of materials, made from recycled waste, and can be used in other similar applications.

## Introduction

Trace elements undergo biogeochemical cycling on the Earth. They are also significantly involved in various biological processes. The role of trace elements and their impact on the environment and living organisms depends not only on their total concentrations but also on the chemical species in which they are actually present. The individual chemical forms of elements determine their toxicity, mobility and bioavailability^1,2^. Management of waste substances is an emerging problem and depends on the characteristics and the content of pollutants in the waste. In order to save natural resources, growing attention is being paid to the efficient reuse and recycling of waste^3–7^, which are the main challenges of the circular economy^8^. When waste cannot be reused or recycled, efforts are focused on adequate treatment for ensuring its safe disposal^9^. To prevent environmental hazards of recycled materials, it is necessary to follow the release of the most mobile, toxic chemical species of elements into the target environmental compartments^3,10–18^. For such studies, speciation analysis has become an indispensable tool^19,20^ as it enables quantitative determination of individual chemical species of trace elements in different sample matrices. The basic analytical tool for the speciation analysis is a combination of a separation technique with element specific detector. Frequently used is high performance liquid chromatography (HPLC) coupled with inductively coupled plasma mass spectrometry (ICP-MS), which is a sensitive, robust and versatile element specific detector. HPLC-ICP-MS was mostly used for speciation of a single element in a given sample^21^. In the environmental studies, when the effects of potentially toxic elements on the environment are investigated, it is necessary to determine the chemical species of multiple elements in the same sample. In this case, the use of single speciation analysis is time-consuming, expensive, and therefore not efficient. Multielemental speciation analysis on the other hand offers the possibility of reducing analyses time and costs, especially in cases, when large numbers of samples need to be analyzed. Despite its multielemental capability, ICP-MS was rarely used as a detector in simultaneous multielemental speciation analysis. This is due to the fact that simultaneous speciation of elements is possible only if the chemical species of elements behave similarly, and are effectively separated on a chromatographic column under the same chromatographic conditions. Marcinkowska et al.^22^ overviewed the applications of multielemental speciation analysis by HPLC hyphenated with ICP-MS. Chemical species of two, three or four elements: arsenic (As), selenium (Se), chromium (Cr), cadmium (Cd), antimony (Sb) and tellurium (Te) were simultaneously determined. These studies were mostly related to environmental water samples. For each set of elements, the chromatographic conditions were optimized and the polyatomic interferences in the ICP-MS determination were eliminated using ammonia or oxygen as the reaction gases, or helium as the collision gas in the dynamic reaction or collision cells^23–28^. Simultaneous speciation analysis was also used to assess the impact of potentially toxic elements on the terrestrial and aquatic environment. To this end, Wolf et al.^29^ studied the influence of forest fires on the mobility and transformation of chemical species of As, Se and Cr in soil. Soil and wildfire ashes leachates were analyzed by applying reversed phase ion-pairing HPLC-ICP-MS procedure for simultaneous speciation of As(III), As(V), Se(IV), Se(VI), Cr(III) and Cr(VI). In our group, a novel analytical procedure was developed for the simultaneous speciation of chromate, molybdate, tungstate and vanadate in alkaline extracts of manual metal arc (MMA) welding fumes loaded on filters^30^. Negatively charged oxyanions were separated on the anion-exchange chromatographic column and detected on-line by ICP-MS. The procedure was further optimized for the simultaneous determination of chromate, molybdate and vanadate in highly alkaline leachates of construction composites prepared from fly ash and cement with the addition of electric arc furnace dust^31^. The obtained results provided complementary information on the release and immobilization of toxic chemical species of elements from construction composites. Drinčić et al.^31^ also developed HPLC-ICP-MS procedure for simultaneous speciation of chromate, arsenate, molybdate and vanadate at alkaline pHs. The separated elemental species were detected by ICP-MS recording masses at the most abundant isotopes at *m/z* 52, 75, 95 and 51, respectively. The advantage of the method is that enables also simultaneous determination of vanadium (V) species in considerably higher concentrations than those of Cr, molybdenum (Mo) and As, using low abundance (0.250%) ^50^V isotope.

To further demonstrate the advantages in using multielemental speciation analysis in environmental studies, the aim of this investigation was to perform simultaneous speciation analysis of chromate (CrO_4_^2−^), molybdate (MoO_4_^2−^) and arsenate (AsO_4_^3−^) in lysimetric water by HPLC-ICP-MS to assess the environmental impacts of geotechnical composites made of different recycled materials installed in field lysimeters.

## Materials and methods

### Instrumentation

Total concentrations of Cr, Mo and As in lysimetric water were determined by ICP-MS, using instrument Agilent 7900 (Tokyo, Japan). Chromate, molybdate and arsenate were separated on an Agilent series 1200 quaternary pump. 7725i Rheodyne injection valve (Cotati, Ca, USA) equipped with a 0.1 mL injection loop was used for sample injection. Separation of elemental species was performed on a strong anion-exchange fast protein liquid chromatography (FPLC) column of Mono Q 5/50 GL (Sigma-Aldrich, St. Luis, MO, USA). To control the stability of the mass spectrometer, the eluent was spiked with internal standards containing 100 ng mL^−1^ of scandium (Sc), germanium (Ge), rhodium (Rh) and indium (In). In speciation analysis, data processing was based on peak area and was processed with Agilent MassHunter software. The ICP-MS operating parameters were optimized for plasma robustness and for the introduction of the minimum amounts of salts used in the separation process using the High Matrix Introduction (HMI) system. To eliminate the polyatomic inferences of chlorine on *m/z* 52 and 75, and carbon on *m/z* 52, the high energy collision mode (HECM) was applied, using helium as a collision gas. ICP-MS operating parameters for total element concentrations and speciation analysis are summarized in Table 1.

**Table 1:** ICP-MS operating parameters.

Parameter	Type/value Speciation analysis	Type/Value Total element concentration analysis
**Sample introduction**		
Nebuliser	Miramist	Miramist
Spray chamber	Scott	Scott
Skimmer and sampler	Ni	Ni
**Plasma conditions**		
Forward power	1550 W	1550 W
Plasma gas flow	15.0 L min^−1^	15.0 L min^−1^
Carrier gas flow	0.75 L min^−1^	1.05 L min^−1^
Dilution gas flow	0.45 L min^−1^	0.00 L min^−1^
He gas flow	10 mL min^−1^	4.5 mL min^−1^
QP bias	− 120 V	− 100 V
Oct bias	− 100 V	− 18 V
Cell entrance	− 150 V	− 38 V
Cell exit	− 150 V	− 62 V
Deflect	− 75 V	− 2.6 V
Plate bias	− 150 V	− 60 V
Sample uptake rate	1.5 mL min^−1^	0.3 mL min^−1^
**Data acquisition parameters**		
*m/z* of isotopes monitored	^52^Cr, ^75^As, ^95^Mo	^52^Cr, ^75^As, ^95^Mo
*m/z* of internal standards	^45^Sc, ^72^Ge, ^103^Rh, ^115^In	^45^Sc, ^72^Ge, ^103^Rh, ^115^In
Total acquisition time	600 s	

The pH was measured with WTW 330 pH meter (WTW GmbH, Weilheim, Germany).

A Mettler AE 163 (Mettler Toledo, Zürich, Switzerland) analytical balance was used for weighing.

### Reagents and materials

Ultrapure 18.2 MΩ cm water (MilliQ) obtained from a Direct-Q 5 Ultrapure water system (Millipore Watertown, MA, USA) was used for the preparation of all solutions. Merck (Darmstadt, Germany) suprapur sodium hydroxide monohydrate (NaOH **·** H_2_O) and suprapur sodium carbonate (Na_2_CO_3_) were used to prepare alkaline buffer solutions (0.2% NaOH + 0.3% sodium carbonate Na_2_CO_3_). Stock standard solutions of Cr, As, Sc, Ge, Rh and In (1000 ± 2 mg L^−1^ in 2–3% HNO_3_) were purchased from Merck. Chromate was prepared from stock solution K_2_CrO_4_ in water (Merck), containing 1000 ± 2 mg L^−1^ of Cr and molybdate from stock solution (NH_4_)_6_Mo_7_O_24_ in water (Merck), containing 1000 ± 2 mg L^−1^ of Mo. Arsenate stock solution (1000 ± 2 mg L^−1^ As) was made by dissolving 0.4170 g of Na_2_HAsO_4_·7H_2_O salt (Sigma-Aldrich, St. Luis, MO, USA) in 100 mL of water. Sodium chloride (suprapur) used in HPLC separations was purchased from Merck. Samples were filtered using 0.45 μm Minisart cellulose nitrate membrane filters (Sartorius, Goettingen, Germany). SPS-SW1 Quality Control Material for Surface Water Analysis purchased from SPS Spectrapure Standards AS (Oslo, Norway), was used to check the accuracy of the total Cr, Mo and As determination in lysimetric water. The determined values for Cr, Mo and As (1.99 ± 0.05 ng mL^−1^, 10.1 ± 0.6 ng mL^−1^ and 9.9 ± 0.2 ng mL^−1^, respectively) agreed well with the certified values (2.00 ± 0.02 ng mL^−1^, 10.0 ± 0.1 ng mL^−1^ and 10.0 ± 0.1 ng mL^−1^). For verifying the accuracy of the determination of Cr(VI) by the HPLC-ICP-MS procedure, Certified Reference Material (Chromium Standard Solution 0.050 mg L^−1^ Cr(VI) ± 0.002 mg L^−1^ Cr(VI) K_2_CrO_4_ in H_2_O) (Merck) was used. A good agreement was obtained between the determined Cr(VI) (46 ± 1 ng mL^−1^) and the certified value (47 ± 2 ng mL^−1^), which confirms the accuracy of the analytical procedure used.

### Preparation of working standard solutions

Multielemental working standard solutions of chromate, molybdate and arsenate used for the speciation analysis were prepared from stock single standard solutions (containing 1000 ± 2 mg L^−1^ of element). First, 0.5 mL of stock standards were pipetted into 10 mL volumetric flasks and filled with MilliQ water to prepare a multielemental standard of chromate, molybdate and arsenate, containing 50.0 ± 0.1 mg L^−1^ of element. Aqueous multielemental standard solutions with concentrations 25.00 ± 0.05 mg L^−1^, 10.00 ± 0.02 mg L^−1^ and 5.00 ± 0.01 mg L^−1^ were prepared from 50.0 ± 0.1 mg L^−1^ standard with appropriate dilution with water. Working standards in an alkaline buffer (0.2% NaOH + 0.3% Na_2_CO_3_, pH 12) in concentrations 50.0 ± 0.5 ng mL^−1^, 100 ± 1 ng mL^−1^, 250 ± 2 ng mL^−1^ and 500 ± 5 ng mL^−1^ were prepared by pipetting 0.1 mL of the appropriate aqueous multielemental standards added to 9.9 mL of an alkaline buffer.

### Analytical procedure for simultaneous speciation of chromate, molybdate and arsenate

The procedure previously developed and validated in our group for simultaneous speciation of oxyanions chromate, arsenate, molybdate and vanadate in water leachates from recycled materials was used^31^. Briefly, 0.1 mL of sample was injected onto the column and linear gradient elution from water to 0.7 M NaCl (0 to 100%) applied for 10 min at a flow rate of 1.5 mL min^−1^. The outlet of the chromatographic column was connected on-line with ICP-MS. After separation, the column was regenerated with 2 mol L^−1^ NaCl for 3 min and equilibrated with water for 7 min. The eluate from the regeneration step was directed to waste through a software controlled six-port valve. Chromatographic program for separation of chromate, molybdate and arsenate on the anion-exchange HPLC Mono Q column is given in Table 2.

**Table 2:** Chromatographic program for separation of chromate, molybdate and arsenate on the anion-exchange HPLC Mono Q column.

Time (min)	Eluent			Flow rate (mL min^−1^)	Steps in the chromatographic procedure
	A (%)	B (%)	C (%)		
0.0	100	0	0	1.5	Separation
10.0	0	100	0	1.5	
10.1	0	0	100	1.5	Regeneration
13.0	0	0	100	1.5	
13.1	100	0	0	1.5	Equilibration
20.0	100	0	0	1.5	

Eluent A: MilliQ water.

Eluent B: 0.7 mol L^−1^ NaCl.

Eluent C: 2 mol L^−1^ NaCl.

If not stated otherwise, all the analyses were done at least in three replicates.

### Lysimetric water

Three geotechnical composites, with commercial names Tersan, Tersan-P and Digeterm, which were composed of recycled waste materials, have been prepared in cooperation with Slovenian mining and waste recycling company Termit d.d. Later on, each of the composites was installed in the field in parallel in two zero-tension box-shaped lysimeters (dimension of each lysimeter was 3 m × 3 m, × 1.2 m), one in uncompacted and the other in compacted form. The lysimeters represented the proxy of an earth structures—geotechnical fills, which are commonly built-in civil engineering with the use of recycled materials. Simulation of a proper installation process, according to geotechnical principles, was performed by compacting each composite in separate lysimeter in 3 layers up to at least 92% of maximum dry density with a handheld vibrating plate compactor. The composites in uncompacted form, were on the other hand filled in the lysimeters without proper compaction process. Therefore, they had approximately 20% lower density. Uncompacted Tersan, Tersan-P and Digeterm also had 1000 times, 100 times and 10 times higher water permeability, respectively, due to higher porosity comparing to their compacted forms. With this experimental approach it was intended to simulate a scenario of what the potential environmental impacts would be if the composites were not installed properly (in line with basic geotechnical demands). The Tersan composite consisted of mining waste (50%), foundry sand (30%) and paper mill sludge (20%), and had a pH of around 8, the Tersan-P composite consisted of mining waste (50%), coal ash (40%), foundry slag (5%) and bottom ash from waste incineration (5%) and had a pH of around 12.5, and the Digeterm composite consisted of digestate from mechanic-biological treatment of municipal waste (40%), paper ash (40%) and mining waste (30%) and had a pH of around 11. For the preparation of geotechnical composites, materials were used as gathered from the industrial processes. Lysimetric water was collected in 1000 L reservoirs after rainfall events. Before analysis, samples were filtered through 0.45 µm filters. Total concentrations of Cr, Mo and As and speciation analysis of chromate, molybdate and asenate in lysimetric waters from uncompacted and compacted Tersan and Tersan-P composites were performed without sample dilution. Since the Digeterm lysimetric water from uncompacted and compacted composites contained high concentrations of dissolved substances, the samples were diluted 5 times before the determinations of the total element concentrations and speciation analysis.

## Results and discussion

### Capability of the HPLC-ICP-MS method for the simultaneous speciation analysis of chromate, molybdate and arsenate

**Figure 1:**
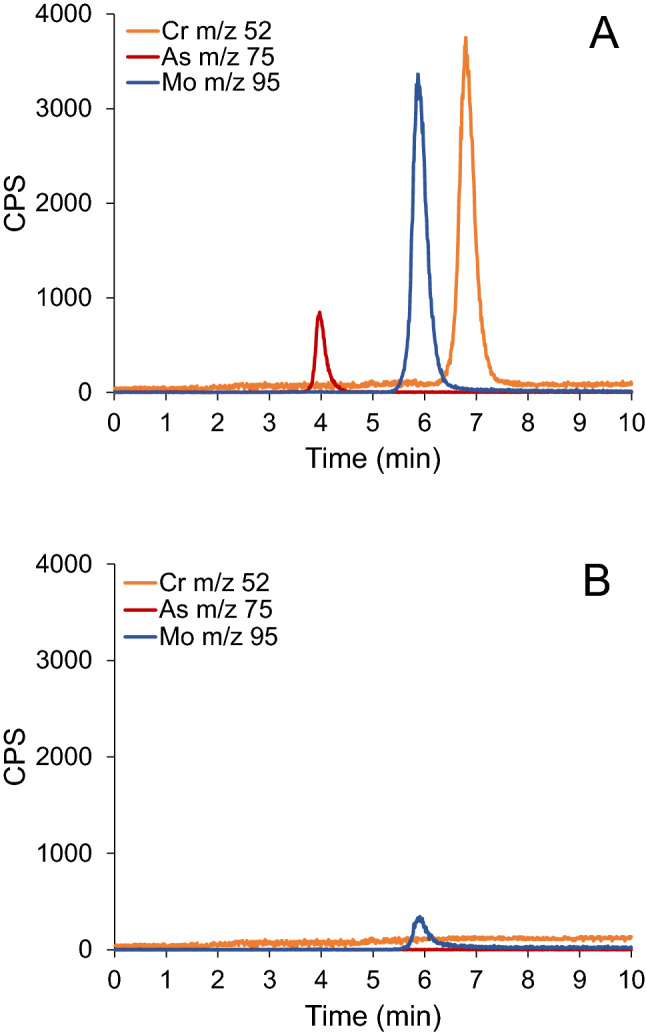
Simultaneous separation of chromate, molybdate and arsenate in 0.2% NaOH + 0.3% Na_2_CO_3_ buffer (pH 12) on the HPLC Mono Q column followed by ICP-MS detection at *m/z* 52, 95 and 75, respectively. (**A**) chromate, molybdate and arsenate (50 ng mL^−1^ of element), (**B**) blank.

To demonstrate the capability of the HPLC-ICP-MS analytical procedure for simultaneous speciation analysis of oxyanions CrO_4_^2−^, MoO_4_^2−^ and AsO_4_^3−^, multielemental working standard solution of oxyanions containing 50 ng mL^−1^ Cr, Mo and As was prepared in alkaline buffer (pH 12) and injected onto the column. The pH of 12 was chosen because these oxyanions are the most stable under highly alkaline conditions. The chromatographic column used enabled the separation at high alkaline conditions^19,30,31^. Separated species were detected simultaneously by ICP-MS recording *m/z* 52, 95 and 75, respectively. Typical chromatograms of multielemental standard solution and blank sample (buffer) are presented in Fig. 1.

As can be seen, arsenate is eluted from 3.8 to 4.3 min, molybdate from 5.4 to 6.6 min and chromate from 6.4 to 7.5 min. Since elution profiles were recorded at different *m/z* by ICP-MS, simultaneous speciation analysis of molybdate and chromate is possible although their peaks are slightly overlapped. The peak areas of the separated oxyanions species (expressed as arbitrary units of counts per second, cps) are 75,800, 61,700 and 10,795 for chromate, molybdate, and arsenate, respectively. This is in agreement with their ionization energies, which are 6.7665 eV, 7.09243 eV and 9.7886 eV for Cr^+^, Mo^+^, As^+^, respectively^32^. Accordingly, the peak area of the eluted As species is the smallest because As^+^ has the highest ionization energy and is less efficiently ionized in plasma than Cr. Blank for Mo originates from metal parts of the pump and represents approximately 9% of the signal of the molybdate standard (50 ng mL^−1^ Mo). In the calculations, the blank for Mo was subtracted from the Mo signal.

The repeatability of measurement was tested for six consecutive simultaneous speciation analyses of a multielemental standard solution of chromate, molybdate and arsenate (50 ng mL^−1^ of element) in alkaline buffer solution (pH 12). The relative standard deviation was found to be ± 0.9%, ± 4.9%, and ± 4.1% for chromate, molybdate, and arsenate, respectively.

Limits of detection (LODs) and limits of quantification (LOQs) were calculated as the concentration that provides a signal (peak area) equal to 3* s* or 10* s* of the blank sample in the chromatogram. To calculate LODs and LOQs, 6 blank samples of the alkaline buffer solution were injected onto the column. LODs for chromate, molybdate and arsenate were found to be 0.16, 0.31 and 0.16 ng mL^−1^, respectively, while LOQs were 0.53, 1.03 and 0.53, respectively. The linearity of measurement for chromate, molybdate and arsenate was obtained over the concentration range from LOQs to 500 ng mL^−1^ with the correlation coefficients better than 0.998.

As no certified reference material is available for the simultaneous determination of chromate, arsenate and molybdate, the accuracy of the analytical procedure was verified by the spike recovery test. For this purpose, multielemental standard solution of chromate, molybdate and arsenate (pH 12), containing 50 ng mL^−1^ of each element, was added to lysimetric water from Tersan-P compacted geotechnical composite. The recoveries were calculated as a ratio between found and added elemental concentration. The results are presented in Table 3.

**Table 3:** Spike recovery test of chromate, molybdate and arsenate for lysimetric water from Tersan-P compacted geotechnical composite spiked with multielemental standard solution of oxyanions containing 50 ng mL^−1^ Cr, Mo and As.

Species	Elemental concentration in lysimetric water (ng mL^−1^)	Elemental concentration added (ng mL^−1^)	Elemental concentration in lysimetric water found (ng mL^−1^)	Recovery (%)
CrO_4_^2-^ *m/z* 52	8.9 ± 0.1	50.0 ± 0.5	58.6 ± 0.6	100
MoO_4_^2−^ *m/z* 95	55.4 ± 2.7	50.0 ± 0.5	109 ± 5	103
AsO_4_^3−^*m/z* 75	< 0.160	50.0 ± 0.5	50.7 ± 2.1	102

Concentrations in the unspiked and spiked samples were determined by simultaneous HPLC-ICP-MS speciation analysis. The results represent the average of three determinations of chromate, molybdate and arsenate with standard deviation of measurements.

Data from Table 3 indicate that recoveries for chromate, arsenate and molybdate of spiked leachate sample lied between 100 and 103%, which confirmed the accuracy of the analytical procedure.

Based on the above described analytical performances, the method was confirmed to be of adequate sensitivity for the simultaneous speciation analysis of oxyanions in lysimetric water.

### Simultaneous speciation analysis of chromate, molybdate and arsenate in lysimetric water

**Figure 2:**
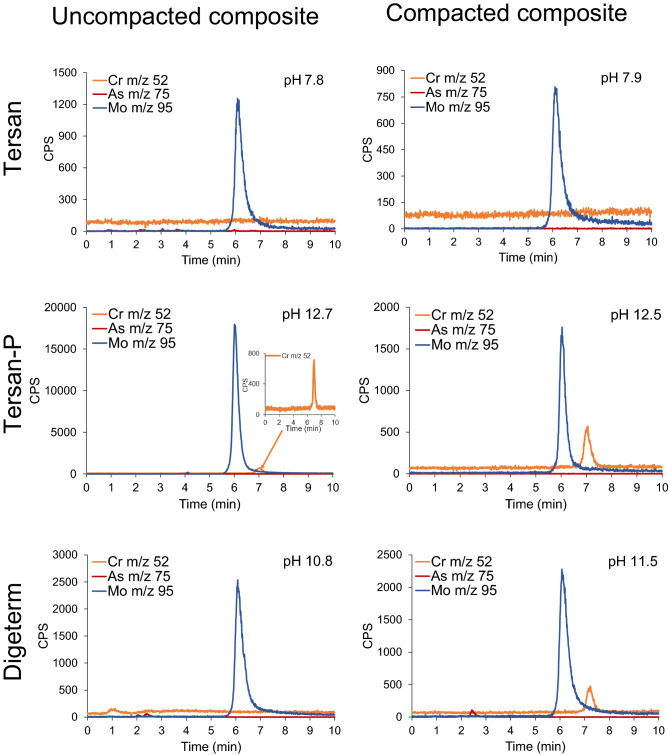
Simultaneous speciation of chromate, molybdate and arsenate in lysimetric water from uncompacted and compacted geotechnical composites, using the HPLC Mono Q column for separation and ICP-MS for detection of separated species at *m/z* 52, 95 and 75, respectively. In Digeterm composites, sample was diluted 5 times before speciation analysis. Total element concentrations and concentrations of chromate, molybdate and arsenate are provided in Table 4.

The simultaneous speciation procedure was used to determine chromate, molybdate and arsenate in lysimetric water from geotechnical composites installed in field lysimeters. Total concentrations of Cr, Mo and As and chromate, molybdate and arsenate in lysimetric waters are presented in Table 4, while the corresponding chromatograms of elemental species in Fig. 2.

**Table 4:** Total concentrations of Cr, Mo and As in lysimetric water from uncompacted and compacted Tersan, Tersan-P and Digeterm geotechnical composites determined by ICP-MS, and concentrations of chromate, molybdate and arsenate determined by HPLC-ICP-MS.

Sample	Total Cr (ng mL^−1^)	CrO_4_^2−^ (expressed as Cr) (ng mL^−1^)	Total Mo (ng mL^−1^)	MoO_4_^2−^ (expressed as Mo) (ng mL^−1^)	Total As (ng mL^−1^)	AsO_4_^3−^ (expressed as As) (ng mL^−1^)
Tersan uncompacted	0.402	< 0.160	56.1	54.8	3.85	< 0.160
Tersan compacted	0.205	< 0.160	38.5	37.5	0.557	< 0.160
Tersan-P uncompacted	10.6	10.3	590	581	1.89	< 0.160
Tersan-P compacted	10.2	8.9	56.8	55.4	0.474	< 0.160
Digeterm uncompacted	80.8	3.33	653	537	36.8	< 0.800
Digeterm compacted	37.7	36.9	627	525	34.6	< 0.800

The results represent the average of three determinations of total Cr, Mo and As concentrations and concentrations of chromate, molybdate and arsenate. Measurement uncertainty for ICP-MS is better than ± 1.5%, while for the HPLC-ICP-MS better than ± 5%.

From data in Table 4 it can be seen that the total As concentrations in lysimetric water from Tersan and Tersan-P composites are low. Lysimetric water from uncompaced Tersan and Tersan-P composites contained 3.9 and 1.9 ng mL^−1^ of total As, respectively, while in lysimetric water from compacted composites, total As concentrations were about 0.5 ng mL^−1^. The data in Table 4 also show that the water percolated from the Digeterm composite contains As in concentration of about 35 ng mL^−1^, from both, the uncompaced and compacted composites. In the environment, inorganic As is present in its trivalent (As(III)) and pentavalent (As(V)) forms. Both, As(III) and As(V) species are toxic, but As(III) is more toxic than As(V)^33^. Speciation analysis (Table 4 and Fig. 2) indicates that arsenate (elution time from 3.8 to 4.3 min) was not detected in any of the analyzed lysimetric water samples from uncompacted and compacted composites. Its concentrations were below LOD, 0.160 ng mL^−1^ in lysimetric water from Tersan and Tersan-P composites, and 0.800 ng mL^−1^ in lysimetric water from Digeterm composites. The higher LOD for Digeterm composites was due to 5 times dilution of samples. The data in Fig. 2 show a small peak of As eluted from 2.4 to 2.7 min in Digeterm lysimetric water from compacted composite, and two small peaks from 2.0 to 2.4 and 2.4 to 2.7 min from uncompacted composite. These As peaks most likely correspond to As complexed by soluble organic matter, which derived from the digestate, one of the main constituents of the Digeterm composite. Currently, there is no legislation regulating on the limit values of contaminants in lysimetric waters. Since the total As concentrations in Tersan and Tersan-P lysimetric water are much lower than those regulated by EU legislation for water intended for human consumption (10 ng mL^−1^ As)^34^, it can be concluded that As in these geotechnical composites was effectively immobilized in compacted and uncompacted form, while in the Digeterm composite, As immobilization was not as effective. However, the risk of groundwater contamination is low.

The data in Fig. 2 and Table 4 further indicate that Mo is eluted as the molybdate anion at concentrations close to the total Mo content in the individual lysimetric waters studied. In the environment, Mo is commonly present in the hexavalent oxidation state (in the form of MoO_3_ and molybdates) and as tetravalent Mo (mainly in the form of molybdenite (MoS_2_)). Mo is an essential micronutrient for plants, animals and humans, while exposure to excessive levels of molybdate is associated with adverse health effects (mostly towards respiratory and renal functions). Mo is widely used in metallurgical applications and is frequently found as environmental pollutant. In the aquatic environment, under physiological conditions (pH > 6.5), Mo compounds are rapidly transformed to MoO_4_^2−^. In low redox environments, molybdate can be reduced to MoS_2_. Mo is more mobile under alkaline conditions, while adsorption increases with decreasing pH. The primary route of human exposure to Mo is food intake and, to a lesser extent, consumption of drinking water^35^. Mo concentration is not regulated in lysimetric water or public drinking water supplies. In line with the need to re-evaluate the latest World Health Organization (WHO) guidelines for drinking water quality, a health-based Mo level of 70 ng mL^−1^ was proposed^36^. As can be seen from the data in Table 4, Mo concentrations in the Tersan lysimetric water with a pH close to 8 are below 70 ng mL^−1^, in the uncompacted composite 54.8 ng mL^−1^ and in the compacted 37.5 ng mL^−1^. The higher Mo content in the lysimetric water from uncompacted composite is due to its less effective physical immobilization associated with a 1000 times higher water permeability compared to the compacted composite. In the Tersan-P lysimetric water with a pH of about 12.5, Mo is highly mobile in uncompacted composite, while in the compacted composite it is effectively immobilized (by physical mechanisms related to 100 times lower water permeability compared to uncompacted composite). In lysimetric water from uncompacted composite, Mo concentrations are 10 times higher than those in water from compacted composite. The behavior of Mo in Tersan-P and Tersan lysimetric water (pH 12.5 and 8, respectively) is consistent with the fact that Mo is more mobile under alkaline conditions, while it is more efficiently adsorbed on the surface of Fe oxyhydroxides at lower pHs^35^. Consequently, its concentration in lysimetric water from uncompacted Tersan composite is significantly lower than in water from uncompacted Tersan-P composite. In Digeterm lysimetric water with high pH (pH around 11), physical immobilization of Mo is not effective. Approximately 530 ng mL^−1^ is leached into lysimetric water from uncompacted and compacted Digeterm composites. Physical immobilization is impaired due to the presence of significant amounts of organic matter derived from the digestate, which is a constituent in the Digeterm composite (40%), making it more porous in both, compacted and uncompacted forms. The concentrations of molybdate in lysimetric water from uncompacted and compacted Tersan (54.8 and 37.5 ng mL^−1^ Mo, respectively), and compacted Tersan-P (55.4 ng mL^−1^ Mo) composites do not represent an environmental hazard. On the other hand, lysimetric water from uncompacted Tersan-P (581 ng mL^−1^ Mo) and uncompacted and compacted Digeterm composites 537 and 525 ng mL^−1^ Mo, respectively) may pose an environmental threat, especially in terms of groundwater contamination with molybdate.

The data in Table 4 and Fig. 2 also show that the total concentration of Cr in the lysimetric water from the uncompacted and compacted Tersan composite was below 0.4 ng mL^−1^ Cr, while no chromate was detected (values were below 0.160 ng mL^−1^ Cr). In lysimetric water from uncompacted and compacted Tersan-P composite, total Cr and chromate concentrations were similar (approximately 10 ng mL^−1^ Cr). In the lysimetric water from compacted Digeterm composite, the content of total Cr and chromate was about 37 ng mL^−1^ Cr. In the water from uncompacted Digeterm composite the total Cr content was significantly higher (81 ng mL^−1^) than the chromate content (3.3 ng mL^−1^ Cr). In the environment, the most stable are trivalent and hexavalent Cr compounds. The latter is highly toxic, carcinogenic and mutagenic^37^. Due to intensive use in different industrial applications, Cr often pollutes the environment. Cr(VI) compounds are highly mobile in environmental compartments and are more stable at alkaline pHs. Cr(VI) is readily reduced by natural reducing agents such as Fe(II), S^2−^ and organic matter. Humans can be exposed to toxic Cr(VI) by consuming contaminated drinking water^21^. Current legislation does not regulate Cr concentrations in lysimetric waters. The total concentrations of Cr in Tersan and Tersan-P lysimetric water are much lower than those regulated by EU legislation for water intended for human consumption (50 ng mL^−1^ Cr)^34^, and WHO guidelines for drinking water quality (50 ng mL^−1^ Cr)^38^. The data in Fig. 2 and Table 4 show that the chromate is effectively immobilized in the compacted Digeterm composite. Concentrations of total Cr and chromate in Digeterm lysimetric waters (approximately 37 ng mL^−1^) are lower than those in the legal requirements for drinking water. In the uncompacted Digeterm composite, the higher porosity of the material (10 times higher water permeability that in the compacted composite) allows the release of soluble organic matter deriving from the decaying organic compounds of the digestate. The released organic matter lowers the pH by almost one unit (pH of lysimetric water in the compacted Digeterm composite was 11.5, while in the uncompacted 10.8). Lower pH and the more efficient contact of Cr(VI) with the organic matter have a favorable effect on its reduction to Cr(III). Of the total Cr concentration 81 ng mL^−1^, only 3.3 ng mL^−1^ remained in its hexavalent form in lysimetric water from the uncompacted Digeterm composite. With regard to toxic chromate concentrations, the investigated lysimetric waters do not represent environmental hazard.

## Conclusions

The potential of simultaneous speciation analysis in studies of the environmental impacts of Cr, Mo and As in geotechnical composites made of different recycled materials installed in field lysimeters was presented.

The results revealed that speciation analysis provides complementary information on the immobilization of Cr, Mo and As and their chemical species in various geotechnical composites, and allows tracking their release in lysimetric water. Arsenate was not present in lysimetric water from uncompacted and compacted Tersan and Tersan-P composites. In Digeterm lysimetric waters, As was most likely complexed by organic matter, while its concentrations (around 37 ng mL^−1^ As) do not represent environmental hazard. Molybdate was detected in all the analyzed samples at concentrations close to the total Mo content. In Tersan (uncompacted and compacted) and Tersan-P compacted composites Mo was leached into lysimetric water in concentrations lower than 56 ng mL^−1^, which are not hazardous to the environment. In Tersan-P (uncompacted) and Digeterm (uncompacted and compacted) composites, Mo was not effectively immobilized and its release into lysimetric water as molybdate in concentrations 525 to 580 ng mL^−1^ Mo, posed an environmental risk for groundwater pollution. Hexavalent Cr was effectively immobilized in all the studied geotechnical composites. Due to the more intensive release of soluble organic matter from the uncompacted Digeterm composite, chromate reduction was very effective in uncompacted composite (total Cr 81 ng mL^−1^, chromate 3.3 ng mL^−1^ Cr), while in the compacted composite the hindered contact with organic matter prevented chromate reduction (total Cr 37.7 ng mL^−1^, chromate 36.9 ng mL^−1^ Cr). However, chromate concentrations released into the lysimetric water from compacted Digiterm composite did not represent environmental hazard. Speciation analysis not only provided data on the presence of toxic elemental species, but also made an important contribution to understanding the physicochemical processes that govern their toxicity. It should be emphasized that simultaneous speciation analysis is a cost-effective analytical tool and allows for rapid analysis of toxic elemental species. Such an analytical approach can be used in many other environmental studies, where a relatively large number of samples need to be analyzed, and for environmental monitoring purposes.

## Data Availability

Data of the current study are available from the corresponding author on reasonable request.

## References

[1] Petruzzelli G, Pedron F, Rosellini I (2020). Bioavailability and bioaccessibility in soil: A short review and a case study. AIMS Environ. Sci..

[2] Crea F, Pettignano A (2020). Chemical speciation of organic and inorganic components of environmental and biological interest in natural fluids: Behaviour, interaction and sequestration. Molecules.

[3] Zalar Serjun V, Mladenovič A, Mirtič B, Meden A, Ščančar J, Milačič R (2015). Recycling of ladle slag in cement composites: Environmental impacts. Waste Manage..

[4] Frías M, García R, de la Villa RV, Martínez-Ramírez S (2016). Coal mining waste as a future eco-efficient supplementary cementing material: Scientific aspects. Recycling.

[5] Rendón-Villalobos R, Ortíz-Sánchez A, Tovar-Sánchez E, Flores-Huicochea E, Poletto M (2016). The Role of Biopolymers in Obtaining Environmentally Friendly Materials. Composites from Renewable and Sustainable Materials.

[6] Tang Z, Li W, Tam VWY, Xue C (2020). Advanced progress in recycling municipal and construction solid wastes for manufacturing sustainable construction materials. Resour. Conserv. Recycl. X.

[7] Zhou C, Wang Y (2020). Recent progress in the conversion of biomass wastes into functional materials for value-added applications. Sci. Technol. Adv. Mater..

[8] van Ewijk S, Stegemann JA (2020). Recognising waste use potential to achieve a circular economy. Waste Manage..

[9] Awuchi CG, Hannington T, Awuchi CG, Igwe VS, Amagwula IO (2020). Industrial waste management, treatment, and health issues: wastewater, solid, and electronic wastes. Eur. Acad. Res..

[10] Maghool F, Arulrajah A, Du Y-J, Horpibulsuk S, Chinkulkijniwat A (2016). Environmental impacts of utilizing waste steel slag aggregates as recycled road construction materials. Clean Technol. Environ. Policy.

[11] Cooper DR, Gutowski TG (2017). The environmental impacts of reuse: A review. J. Ind. Ecol..

[12] Mladenovič A, Hamler S, Zupančič N (2017). Environmental characterisation of sewage sludge/paper ash-based composites in relation to their possible use in civil engineering. Environ. Sci. Pollut. Res..

[13] Oehmig WN, Roessler J, Saleh AM, Clavier KA, Ferraro CC, Townsend TG (2021). Comparison of trace element mobility from MSWI ash before and after plasma vitrification. Waste Manag. Res..

[14] Đurić M, Oprčkal P, Zalar Serjun V, Mauko Pranjić A, Ščančar J, Milačič R, Mladenovič A (2021). Environmental impacts and immobilization mechanisms of cadmium, lead and zinc in geotechnical composites made from contaminated soil and paper-ash. Appl. Sci..

[15] Oprčkal P, Mladenovič A, Zupančič N, Ščančar J, Milačič R, Zalar Sejrun V (2021). Remediation of contaminated soil by red mud and paper ash. J. Clean. Prod..

[16] Saleh TA, Tuzen M, Sari A (2017). Magnetic activated carbon loaded with tungsten oxide nanoparticles for aluminum removal from waters. J. Environ. Chem. Eng..

[17] Saleh TA (2021). Protocols for synthesis of nanomaterials, polymers, and green materials as adsorbents for water treatment technologies. Environ. Technol. Innov..

[18] Saleh TA, Mustaqeem M, Khaled M (2022). Water treatment technologies in removing heavy metal ions from wastewater: A review. Environ. Nanotechnol. Monit. Manag..

[19] Drinčić A, Nikolić I, Zuliani T, Milačič R, Ščančar J (2017). Long-term environmental impacts of building composites containing waste materials: Evaluation of the leaching protocols. Waste Manage..

[20] Llaver M, Fiorentini EF, Oviedo MN, Quintas PM, Wuilloud RG (2021). Elemental speciation analysis in environmental studies: Latest trends and ecological impact. Review. Int. J. Environ. Res. Public Health.

[21] Ščančar J, Milačič R (2014). A critical overview of Cr speciation analysis based on high performance liquid chromatography and spectrometric techniques. J. Anal. At. Spectrom..

[22] Marcinkowska M, Barałkiewicz D (2016). Multielemental speciation analysis by advanced hyphenated technique – HPLC/ICP-MS: A review. Talanta.

[23] Urbánková K, Moos M, Machát J, Sommer L (2011). Simultaneous determination of inorganic arsenic, antimony, selenium and tellurium by ICP-MS in environmental waters using SPE preconcentration on modified silica. J. Environ. Anal. Chem..

[24] Jabłońska-Czapla M, Szopa S, Grygoyć K, Łyko A, Michalski R (2014). Development and validation of HPLC–ICP-MS method for the determination inorganic Cr, As and Sb speciation forms and its application for Pławniowice reservoir (Poland) water and bottom sediments variability study. Talanta.

[25] Marcinkowska M, Komorowicz I, Barałkiewicz D (2015). Study on multielemental speciation analysis of Cr(VI), As(III) and As(V) in water by advanced hyphenated technique HPLC/ICP-DRC-MS. Fast and reliable procedures. Talanta.

[26] Marcinkowska M, Komorowicz I, Barałkiewicz D (2016). New procedure for multielemental speciation analysis of five toxic species: As(III), As(V), Cr(VI), Sb(III) and Sb(V) in drinking water samples by advanced hyphenated technique HPLC/ICP-DRC-MS. Anal. Chim. Acta.

[27] Michalski R, Szopa S (2015). Simultaneous determination of inorganic forms of arsenic, antimony, and thallium by HPLC-ICP-MS. Spectroscopy.

[28] Sun J, Yang Z, Lee H, Wang L (2015). Simultaneous speciation and determination of arsenic, chromium and cadmium in water samples by high performance liquid chromatography with inductively coupled plasma mass spectrometry. Anal. Methods.

[29] Wolf RE, Morman SA, Hageman PL, Hoefen TM, Plumlee GS (2011). Simultaneous speciation of arsenic, selenium, and chromium: Species stability, sample preservation, and analysis of ash and soil leachates. Anal. Bioanal. Chem..

[30] Ščančar J, Berlinger B, Thomassen Y, Milačič R (2015). Simultaneous speciation analysis of chromate, molybdate, tungstate and vanadate in welding fume alkaline extracts by HPLC-ICP-MS. Talanta.

[31] Drinčić A, Ščančar J, Zuliani T, Nikolić I, Milačič R (2017). Simultaneous speciation of chromate, arsenate, molybdate and vanadate in alkaline samples by HPLC-ICP-MS at different concentration levels of vanadate. J. Anal. At. Spectrom..

[32] Lide DL (1998). CRC Handbook of Chemistry and Physics.

[33] Nurchi VA, Buha Djordjevic A, Crisponi G, Alexander J, Bjørklund G, Aaseth J (2020). Arsenic toxicity: Molecular targets and therapeutic agents. Biomolecules.

[34] Official Journal of the European Communities, 2020. Directive (EU) 2020/2184 of the European Parliament and of the Council of 16 December 2020 on the quality of water intended for human consumption (recast).

[35] Todd, G.D., Ingerman, L., Keith, S., Citra, M., O. Faroon, Diamond, G.L., Ph.D. Buser, M., Hard, C., Klotzbach, J.M., Nguyen, A. 2020. Toxicological profile for molybdenum. Agency for Toxic Substances and Disease Registry, U.S. Department for Health and Human Services, https://www.atsdr.cdc.gov/toxprofiles/tp212.pdf (last accessed on 29.4.2022).

[36] Frisbie SH, Mitchell EJ, Sarkar B (2015). Urgent need to reevaluate the latest World Health Organization guidelines for toxic inorganic substances in drinking water. Environ. Health.

[37] DesMarias TLT, Costa M (2019). Mechanisms of chromium-induced toxicity. Curr. Opin. Toxicol..

[38] World Health Organization. (2011). Guidelines for drinking-water quality, 4th edition, http://whqlibdoc.who.int/publications/2011/9789241548151_eng.pdf. Last accessed on 27.04.2022.

